# Supplementary material for: TFOFinder: Python program for identifying purine-only double-stranded stretches in the predicted secondary structure(s) of RNA targets
Source: PLoS Comput Biol. 2023 Aug 25;19(8):e1011418. doi: 10.1371/journal.pcbi.1011418 (PMC10484449; doi:10.1371/journal.pcbi.1011418)
Supplement: S1 Text — (PDF) [file pcbi.1011418.s002.pdf]

S1 Text. Example of descriptors used for the *RNAMotif* searches. The content of individual descriptor files is provided within quote symbols (“”) after a short description.

1. Double stranded A12 search - one strand of target (the 2nd line should be removed if both strands need to be searched).

“parms
chk_both_strs=0;

descr

h5(len=12, seq="AAAAAAAAAAAA")

ss (maxlen = 100000)

h3”

2. Double-stranded R12 search – one strand of target

“parms
chk_both_strs=0;

descr

h5 (len = 12, seq="^r*$")

ss (minlen = 0, maxlen = 100000)

h3”

3. Double-stranded R12 with GU base pairs (R12_GU) – one strand of target

“parms
chk_both_strs=0;

wc += gu;

descr

h5(len=12, seq="^r*gr*$")

ss(minlen=0, maxlen=100000)

h3”

4. Double-stranded R12 with one mispair (R12_1MP) – one strand of target

“parms
chk_both_strs=0;

descr

h5(len=12, seq="^r*gr*$", mispair=1)

ss(minlen=0, maxlen=100000)

h3”

5. Double-stranded R11Y (one pyrimidine) – one strand of target

“parms
chk_both_strs=0;

descr

h5 (len = 12, seq="^rr*yr*r$")

ss (minlen = 0, maxlen = 100000)

h3”

6. Double-stranded R10Y2 (two pyrimidines strict) – one strand of target

“parms
chk_both_strs=0;

descr

h5 (len= 12, seq="^r\{1,8\}y\{1\}r\{1,8\}y\{1\}r*r\{1\}$")

ss (minlen = 0, maxlen = 100000)

h3”

7. Double-stranded R10Y2 (two pyrimidines relaxed) – one strand of target

“parms
chk_both_strs=0;

descr

h5 (len= 12, seq="^r*y\{1\}r*y\{1\}r*$")

ss (minlen = 0, maxlen = 100000)

h3”
