# Supplementary material for: TFOFinder: Python program for identifying purine-only double-stranded stretches in the predicted secondary structure(s) of RNA targets
Source: PLoS Comput Biol. 2023 Aug 25;19(8):e1011418. doi: 10.1371/journal.pcbi.1011418 (PMC10484449; doi:10.1371/journal.pcbi.1011418)
Supplement: Attachment — Submitted filename: Response_PCOMPBIOL-D-23-00670.pdf [file pcbi.1011418.s003.pdf]

Yeshiva University

August 8th, 2023

Dear Dr. MacKerell and Dr. Noble,

Thank you very much for the thorough reviews of our manuscript. For the past few weeks, we have been working to address the reviewers' comments. Below we describe in detail the revisions we made to our manuscript in order to meet their requests, as follows:

- We included the reviewers' comments and highlighted them with red font.
- Our responses are enclosed in >> … << marks.
- The revised text is included in this letter under quotation marks, and it is accompanied by the corresponding line numbers in the clean and tracked versions of the revised manuscript.
- We inserted the "Author Summary" paragraph only in the clean version of the manuscript (docx format).
- We removed the figures from the clean file (docx format) but not from the tracked file.
- We uploaded the figures as individual tif files.

In addition, we are including one pdf copy and one docx source file of the manuscript:

- PCOMBIOL-D-23-00670_with_tracking_071223.pdf file reflects all changes we made to our original submission as follows:
    - Insertions
    - ~~Deletions~~
    - Changed lines = grey outside border
    - ~~Moved from~~
    - Moved to
    - Change format = side bubble
- PCOMBIOL-D-23-00670_clean_071223reftxt.docx file is the clean version of the revised manuscript.

We would also like to thank the reviewers for their time and valuable assessment.

Sincerely,

Atara Neugroschl and Irina Catrina

Comments to the Authors:
Please note here if the review is uploaded as an attachment.

Reviewer #1: Neugroschl and Catrina describe TFOFinder, their Python program that identifies intramolecular purine-only RNA duplexes that are amenable to forming parallel triple helices. They used this program to analyze the Drosophila genome for potential triplex target sites. The manuscript appears to be well-written. In my opinion, while the tool may be helpful for a small group of scientists interested in targeting triplex sites, this appears to be a simple, incremental step that won't have much impact on the field.

>>While specialized expertise may be needed to experimentally apply our software, we believe that *TFOFinder* can greatly reduce the time needed to design these probes manually when including MFE (minimum free energy) and SO (suboptimal) structures and we hope that it will encourage studies that will expand the application of triple helixes beyond this small area of expertise. We are happy to address all comments as detailed below. <<

The authors may want to consider the following:
• Perhaps an explanation of how RNAMotif and TFOFinder differ, how they can be used in conjunction, and how TFOFinder advances the field would be beneficial for the readership.

>>The *RNAMotif* and *TFOFinder* programs are not redundant; *RNAmotif* can be used to identify potential RNA targets in a large dataset, which then can be analyzed using *TFOFinder*. However, while *RNAmotif* can be used to identify any regions that may contain a purine duplex in RNA targets of interest, it does not take into consideration the target structure.

This was briefly discussed in the manuscript, and at the Reviewer #1's recommendation we expanded this section to include more information in the paragraph first introducing *TFOFinder*, as shown below:

Lines clean file 170-176 & tracking file 153-159: "The *TFOFinder* program takes into consideration the predicted secondary structure(s) of an RNA target of interest and designs the corresponding TFO probes, features that are not implemented in *RNAmotif*. However, when large-scale transcriptome-wide studies are performed, *RNAmotif* is an invaluable tool for first identifying RNA sequences that show the potential to form purine duplexes. These results can be further analyzed using *TFOFinder*; to include structure information obtained using freely available RNA folding software (*e.g.*, *mfold* [34]; reviewed in [35]) and design TFO probes."

In addition, we included several examples of *RNAmotif* descriptor files as supporting information and referenced them under the Methods section:

Line clean file 491 & tracking file 496: "**S2 Text. Example of descriptors used for the *RNAmotif* searches.**" <<

• Line 67, “therapeutics approaches” should be “therapeutic approaches.”

>> Line clean file 84 & tracking file 67: This was corrected as indicated. <<

• Line 77, what is meant by “frame”? Backbone?

>> Line clean file 94 & tracking file 77: Yes, “frame” here means backbone, and we did change it, as the reviewer implied it will make it clearer. <<

• Line 81, “in a greater mismatch discrimination” should be “with a greater mismatch discrimination.”

>> Line clean file 98 & tracking file 81: This was corrected as indicated. <<

• Line 100, “duple-formation” should be “duplex-formation.”

>> Line clean file 117 & tracking file 100: This was corrected as indicated. <<

• Line 134, FRET should be defined.

>> Line clean file 144 & tracking file 127: This was defined in the text only, but it was not included in the list of abbreviations, as it only appears once.

“FRET (Fluorescence/Förster Resonance Energy Transfer)” <<

• Line 133, this paragraph seems to be out of place. The authors provided introductory material and then introduced their new tool. This paragraph of introductory material sits between two paragraphs that discuss the tool. Is there a better location in the introduction to move this paragraph?

>> Lines clean file 143-152 & tracking file 126-135: This paragraph was moved just above the previous paragraph, which introduces *TFOFinder*.<<

• Line 259, MFE and SO are used but not defined until lines 374-375.

>> Line clean file 284 & tracking file 287: The definitions were moved where the MFE and SO first occur in the text, as requested. <<

• Line 298, what is sscount?

>> <u>Lines clean file 324, 329-338 & tracking file 327, 332-341:</u> We added a reference for this number, which we defined in a previous publication (reference [47]) and we included the following information immediately following the sentence first mentioning "ss-count":

"The ss-count fraction indicates the extent to which a sequence is predicted to be single-stranded in the predicted MFE and/or SO structures. The larger the value of the ss-count fraction, the more likely it will be that the sequence will have a single-stranded character, where 1 = fully single-stranded and 0 = fully double-stranded. The ss-count fraction was calculated by dividing the sum of the ss-count numbers of the individual bases in the TTS to the product of the probe length and number of total structures (MFE and SO structures) in the input file. The ss-count number represents the number of structures of the total structures in which a base is predicted to be single-stranded, and the ss-count file is one of the output files obtained when predicting RNA structure using *mfold*." <<

- Line 309, "one highlighted in the red box for the of the 12th 310 SO structure" does not make sense and needs editing.

>> <u>Lines clean file 340-345 & tracking file 343-349:</u> The legend of Fig 5 was edited as follows:

"**Fig 5. Secondary structures for two ncRNAs, predicted with *mfold*.**

(A) Full MFE structure (*mfold*) of the shortest transcript (*CR44598-RA*) identified to contain one TTS, which is highlighted in the red box and also shown magnified (right). (B) A longer ncRNA (*CR44619-RA*, 1,023-nt) containing several TTS, and one TTS containing a mispair and 1-nt bulge in the MFE structure (left, red arrows) is highlighted in the red boxes for the MFE and 12th SO structure (*mfold*; right)." <<

- Line 377, define NCBI.

>><u>Line clean file 404 & tracking file 409:</u> This was defined in the text at the first mention, but it was not included in the list of abbreviations, as it only appears three times in the manuscript.

"NCBI (National Center for Biotechnology Information)" <<

• Ref 14 doesn't look complete.

>>Lines clean file 540-543 & tracking file 560-563: "14. Gupta P, Zengeya T, Rozners E. Triple helical recognition of pyrimidine inversions in polypurine tracts of RNA by nucleobase-modified PNA. Chem Commun (Camb). 2011;47(39):11125-7. doi: 10.1039/c1cc14706d. PubMed PMID: 21909545; PubMed Central PMCID: PMCPMC3757498."

At the reviewer's request, we checked the above reference and found that it was imported in EndNote using the citation file provided by the Journal and we believe it is complete. If the reviewer has a specific observation regarding this reference, we would be happy to check it again. <<

Reviewer #2: General Comment

The authors have presented a potentially valuable and innovative tool for designing TFOs targeting RNA in the model species D. melanogaster (genome and transcriptome) and the vRNA8 of influenza A. They have searched for double-stranded fragments of a user-defined length (4-30 nt) composed of consecutive purines within predicted secondary structures of the RNA target of interest.

The literature review and description of the methods employed by the authors are clear and concise, and the rationale for the study is evident. We appreciate the authors providing the link to the Github repository containing the TFOFinder python code.

While we believe that the wider scientific and bioinformatics community can benefit from this work, we suggest the authors consider applying the FAIR (Findable, Accessible, Interoperable, and Reusable) principles to the manuscript to ensure reproducibility and reusability of the codebase. It would be helpful if the authors could provide test data to demonstrate the usage of the provided scripts and how they integrate with other tools used in the complete study.

>>We apologize for not including examples with our scripts. At the Reviewer #2's recommendation we updated the GitHub repository to include examples of *TFOFinder* input and output files for the 67th RNA identified by our *D. melanogaster* transcriptome search (*ove-RE* mRNA, S1 Table). We also wrote and uploaded in GitHub a tutorial file describing in detail the *TFOFinder* example mentioned above. We will make available upon request any of the files mentioned in our manuscript.

The GitHub repository was updated by making a new folder for all *TFOFinder*-related files, and the GitHub link was updated, and the following text was included in the revised manuscript:

Line clean file 463 & tracking file 468: The link to the GitHub repository was updated: "(https://github.com/icatrina/TFOFinder)"

Lines clean file 472-475 and tracking file 477-480: "A tutorial file can be found in the above-

mentioned GitHub repository. This tutorial provides details for the download and installation

requirements, as well as the usage of *TFOFinder* for the 67th RNA target, *ovo-RE* mRNA (S1 Table). The input and output files for this example are also provided.”

Additionally, clearer documentation of the code is necessary to further enhance the credibility of the study.

>>The GitHub repository was also updated to include the following files:

- GNU_GPL.txt license file
- Updated README file
- Installation notes
- Tutorial file (use download button to view this file)
- Example of an input file – (4.13MB size file that cannot be viewed directly in GitHub; 67_ovo_RE.ct)
- Example output files corresponding to the above-mentioned input file (67_ovo_RE_TFO_probes.txt and 67_ovo_RE_sscount.csv)

The application of TFOFinder for identifying conserved TTS in the influenza A virus was a notable achievement. Based on their analysis, we wonder if the authors are considering conducting further studies on RNA targets of other respiratory viruses or perhaps viruses that affect plants?

>>Thank you for your comment, and yes, we are planning to analyze other viruses, and following your kind suggestion we will include plant viruses as well. <<
